# Surface Electromyographic Activity of the Rectus Abdominis and External Oblique during Isometric and Dynamic Exercises

**DOI:** 10.3390/jfmk7030067

**Published:** 2022-09-06

**Authors:** Athanasios Mandroukas, Yiannis Michailidis, Angelos E. Kyranoudis, Kosmas Christoulas, Thomas Metaxas

**Affiliations:** 1 Laboratory of Evaluation of Human Biological Performance, Department of Physical Education and Sport Sciences, Aristotle University of Thessaloniki, 57001 Thessaloniki, Greece; 2 Department of Physical Education and Sport Sciences, Democritus University of Thrace, University Campus, 69100 Komotini, Greece

**Keywords:** abdominal muscles, electromyography, isometric, concentric, eccentric, strength exercises

## Abstract

Exercises for the abdominal muscles are widely used in athletic activities for strength training and prevention of low back disorders. The timing and volume of muscle activation for various movements have not yet been clarified. The purpose of this research was to evaluate the surface electromyographic activity from the upper (RAU) and lower part (RAL) of the rectus abdominis and the external oblique (EO) muscles during eleven abdominal muscle strength training exercises. Thirty-five healthy male university students with a subspecialty in soccer participated in the study. They performed exercises involving isometric, concentric, and eccentric muscle contractions. The electromyographic recordings were amplified by a factor of 1000, rectified, and integrated. Mean integrated values were calculated by dividing the elapsed time for the five repetitions, to provide the mean integrated electromyographic values for each exercise. Leg movements from a long lying position showed higher activity of the rectus abdominis, compared to the EO (*p* < 0.001). The dynamic sit-ups showed an obvious dominant activity of the EO, compared to the rectus abdominis (*p* < 0.001). During eccentric exercise, higher activity was found in the RAU, compared to the RAL (*p* < 0.001). The results could serve as a basis for improving the design and specification of training exercises. Pre-exercises should be performed before testing abdominal muscle strength.

## 1. Introduction

The importance of abdominal muscle strength is well recognized for the prevention of lower back disorders and the proper execution of occupational and athletic activities, and for this reason special exercises are used for its development [1,2,3,4,5]. Surface electromyography provides clear information about the measurements of various muscle activation patterns, which should be taken into account when selecting and prescribing strengthening exercises, because the force of the muscle contraction is regulated by the total amount of motor units recruited [6,7,8]. The relative activation of the abdominal muscles in various forms of training exercise has been adequately explored. In many exercises the abdominal muscles have a stability function, so that a given movement is facilitated and can be well performed.

Frequently during training, the flexor muscles of the hip participate more than it is necessary and as a result, the abdominal muscles are less activated [9]. The abdominal muscles have a phasic function, and when an activation is either incomplete or wrong, they tend to weaken and are less activated [10]. It is known that training of the trunk and pelvic muscles is a necessary condition for the stabilization and support of the spinal column [11]. This becomes particularly evident during fast and extreme movements of the body. In some fundamental body movements such as walking, running, jumping, etc., the pelvis and the lumbar spine constitute a central functional unit. The pelvis, connected to the sacrum and located in a neutral position, maintains the normal curvature of the spine.

A strong muscle corset around the lumbar spine increases stability, prevents improper loading of the spine, contributes to unloading of the erector spinae, and thus also reduces the compression force on the intervertebral discs of the lumbar spine [12,13,14,15,16]. It is generally accepted that testing and training of the abdominal muscles should be done with curl-ups, performed from the long lying or hook lying positions. In both cases the curl-ups begin with flexion of the head, while the shoulders peel up with a rounded back to approximately 35–40° from the floor, so that the lumbar spine remains on the floor. It is recommended that the curl-up should be performed with flexed unsupported knees, without holding the knees or feet, and then slowly curling up with a rounded back to 35–40°, without trunk flexion. The reasons for this are (1) to avoid the uneven loading on the lumbar spine, and (2) to isolate the activity of the hip flexors. The stress placed on the lumbar spine decreases by limiting the amount of trunk flexion to 35–40° [17]. Therefore, curl-ups performed through a partial range may be an effective method of gaining abdominal muscle strength, while protecting the lumbar spine.

To perform trunk flexion or curl-up with straight or bent knees to a fully upright seated position, the back muscles must have a normal length [18]. Otherwise, the participant may be unable to perform the curl-up correctly, despite sufficient strength of the abdominal muscles. The time and volume of activation in a movement, as well as the functional relationship between the abdominal and back muscles, have not yet been clarified. Furthermore, there have been no studies comparing these kind of exercises in terms of electromyographic activity. The purpose of the study is to investigate the activation of the upper portion of the rectus abdominis (RAU), the lower portion of the rectus abdominis (RAL), and the external oblique (EO) in various patterns of isometric and dynamic exercises, in order to obtain further knowledge on the recruitment of these muscles, which can be applied during proper physical training.

## 2. Materials and Methods

### 2.1. Study Design

This study examined eleven exercises that are often used for training of the abdominal muscles. The investigated exercises included dynamic exercises (concentric and eccentric) and isometric contractions of the abdominal muscles. A rest period of approximately 2–3 min was given between each exercise. If a subject did not perform the exercise according to the instructions, the trial was repeated.

### 2.2. Participants

The calculations for effect size (ES) and statistical power were performed with G*Power software: Statistical Power Analyzes for Windows, Version 3.1.9.7 [19,20], according to Cohen’s criteria [21,22]. The power analysis was conducted prior to the study being performed, based on previous studies of similar research design. An effect size of >0.25, a probability error of 0.05, and a power of 0.95 were used for the present research. These indicated that 27 subjects comprised the smallest acceptable number of participants for the analysis.

Surface electromyographic (EMG) recordings were generated from 35 healthy male university students with a subspecialty in soccer (mean age 22.5 ± 1.9 yrs; training experience 12.6 ± 2.1 yrs; height 176.5 ± 3.8 cm; weight 72.0 ±4.3 kg). All participants were informed of the nature, purpose, procedures, potential discomfort, risks, and benefits involved in the study before giving their written consent for participation. None of the participants had undertaken progressive resistive exercise 24 h prior to the testing, and their sleep patterns were sufficient (approximately 8 h) in order to arrive at the laboratory in a rested condition. All training sessions and measurements were conducted at the same day and time, under the same conditions. All participants completed a questionnaire that included their relevant medical and physical history. Three people were excluded from the mobility tests and pre-exercises, because they could not meet the technical execution of the movements according to the instructions. The subjects typically trained 3 to 4 times per week and participated in university soccer competition. They were healthy with no back pain, and volunteered to participate in the study. They were free of overly extensive adipose tissue in the abdominal region. Participants were instructed to perform exercises that are frequently used in rehabilitation and athletic programs. Each participant was given instructions for each exercise prior to testing, and none had consistently trained with stabilization exercises previously. The exercises were performed in the same order as they are presented in this paper, as shown in the numbered figures which include test results. If a subject did not perform the exercise according to the instructions, the test was repeated. This study has been approved by the Institutional Review Board of the Exercise Physiology and Sport Rehabilitation Laboratory, Thessaloniki, Greece (No. 01/2021), and was in accordance with the Declaration of Helsinki.

### 2.3. Mobility Tests and Pre-Exercises for the Correct Execution of the Movements

**Figure 1:**
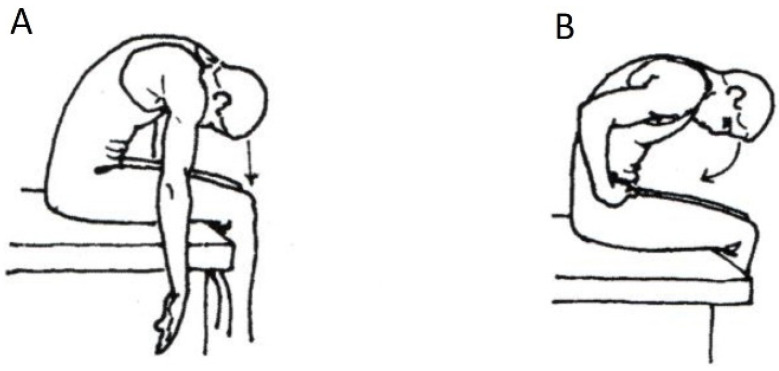
(**A**) Mobility test of the trunk with flexion of the head and pelvic tilt (rotation), and (**B**) without the pelvis tilted forward.

During the instructions and the execution of the exercises, it was ascertained that, despite the general uncontrolled (i.e., without guidance) training that followed, the participants lacked proper mind–muscle connection, and coordination of the complex movements involved, and they demonstrated intense muscular shortening in the iliopsoas muscle, in the adductors, and in the hamstring muscles. Thus, two tests were performed to determine the mobility of the back muscles (spinal flexibility) to ensure that limited mobility did not affect the complete flexion of the trunk; and to achieve the correct movement in the curl-up. The subjects were instructed to sit on a high seat or plinth, with the trunk in an upright position (Figure 1). This starting position was chosen to eliminate any involvement of the hamstring muscles. From this initial position the participants slowly flexed their head, followed by the cervical, thoracic, and lumbar spine, after which the pelvis was tilted (Figure 1A). The participants were trying to touch their forehead to their knees, with a distance between 10–15 cm considered as normal. The distance was measured with a tape measure; if there was shortening in the back muscles, the expected distance would not be attained. In this test, the mobility of the spine was measured from C7-S1. In the second, more specialized test (Figure 1B), the subjects stabilized the pelvis with their hands without the pelvis tilted forward. In this test, the mobility of the lumbar spine was measured from S1 and 10 cm upwards, where this distance normally increased by 4–5 cm in the final phase of the movement.

**Figure 2:**
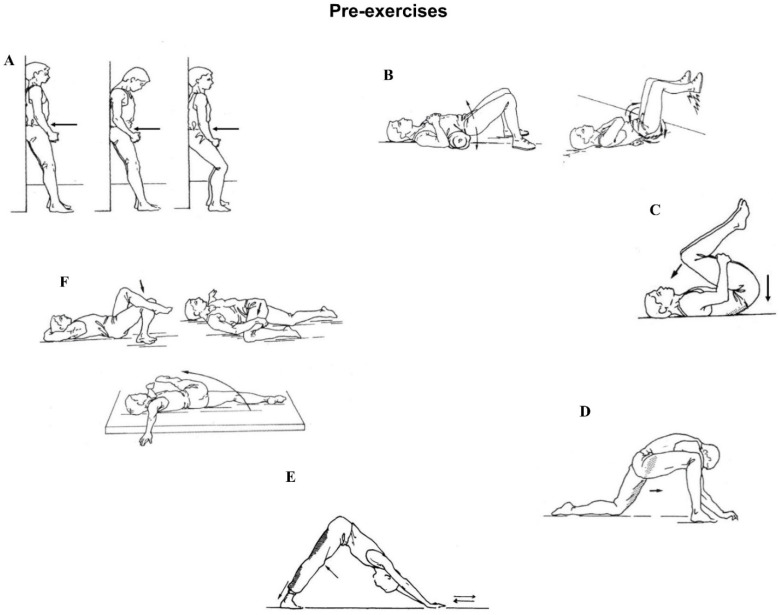
Pre-exercises that were performed prior to the testing procedure. (**A**): Pelvic tilting in upright position by flattening back and sliding up and down against a wall, to initiate the concept of proper bending and squatting. (**B**): Gentle rotation and elevation of the pelvis forward and backward. Feet on the floor and wall. (**C**): Both knees towards the chest into a fully flexed position, the lumbar spine pressed to the floor. (**D**): Stretching the iliopsoas muscle. (**E**): Stretching the hamstrings and gastrocnemii muscles. (**F**): Stretching the obliqui and muscles around the pelvis.

It was considered necessary to introduce the pre-exercises (Figure 2) before performing the exercises using the EMG.

### 2.4. Investigated Exercises

**Figure 3:**
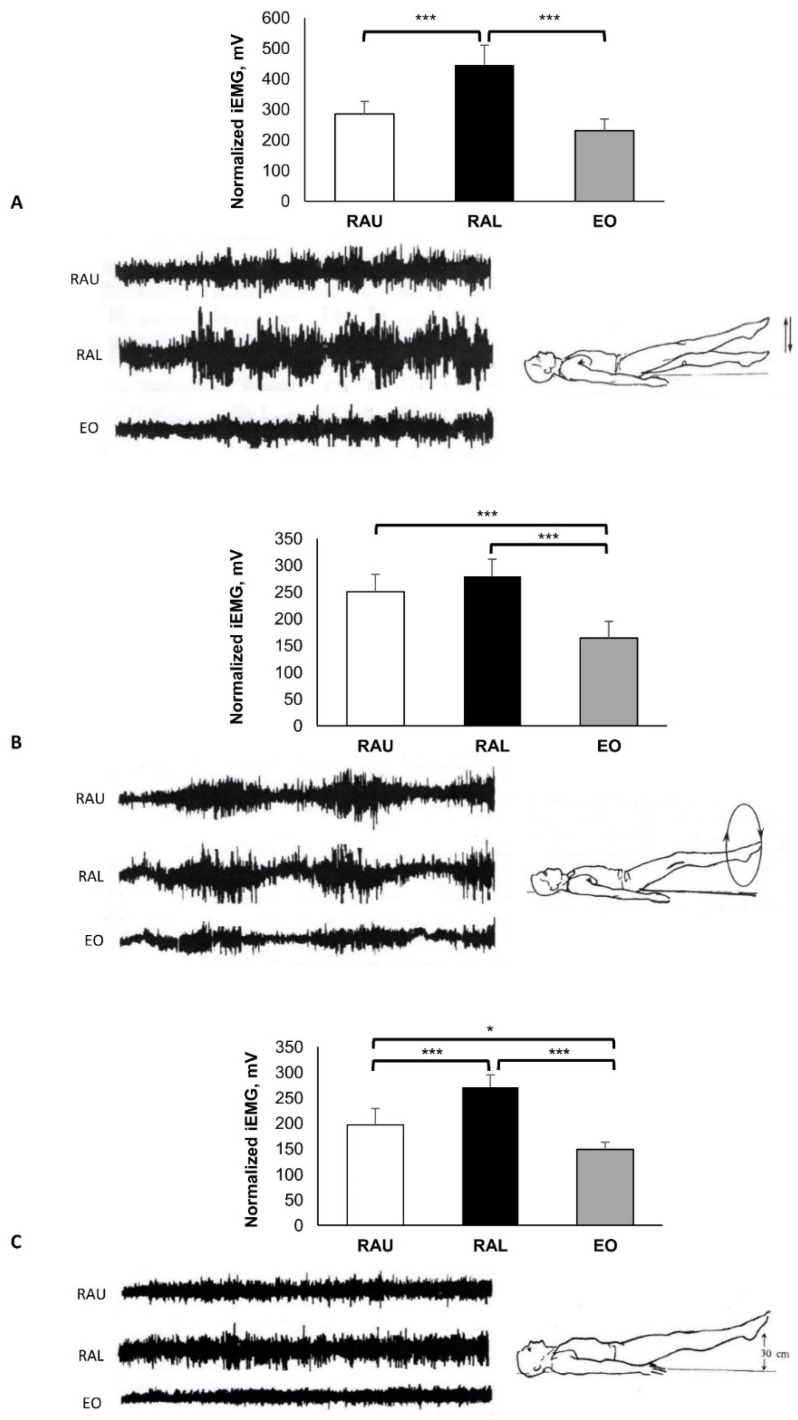
Long lying. Dynamic exercises: (**A**) Scissors exercise, (**B**) in circles, and (**C**) straight legs rising 30 cm from the floor with isometric contraction for 10 s (**C**). Group mean electric activity during the exercise. iEMG indicates surface electric activity. Raw EMG activity of the three muscles from one participant. RAU = rectus abdominis upper part; RAL = rectus abdominis lower part; EO = external oblique. Differences between muscles: * *p* < 0.05; *** *p* < 0.001).

**Figure 4:**
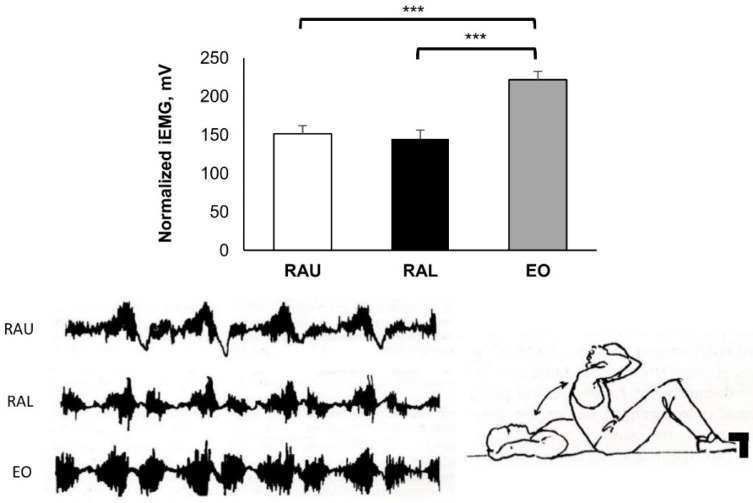
Hook lying with bent knees and support on the feet. Quick start; flexion and extension of the trunk five times. Group mean electric activity during the exercise. iEMG indicates surface electric activity. Raw EMG activity of the three muscles from one participant. RAU = rectus abdominis upper part; RAL = rectus abdominis lower part; EO = external oblique. Differences between muscles: *** *p* < 0.001.

In all exercises, the subjects were lying in the supine position on the floor. All exercises were performed following the same sequence and under the supervision and guidance of an experienced physiotherapist. The dynamic exercises (Figure 3A,B and Figure 4) were performed with five continuous repetitions, with the pace set by a metronome (50 b/min).

Figure 3: Starting position: Long lying. Straight bilateral legs moved up and down in scissors (A) and in circles ten times (B). Straight leg rising 30 cm from the heels to the floor and isometric contraction for 10 s (C). The instructions to the subjects during these exercises were to maintain the pelvis in a neutral position.

Figure 4: Starting position: Hook lying with bent knees (110°), support on the feet and the hands behind the neck. Full sit-ups (ten times) with quick start from the floor. During the sit-ups the elbows came inward, towards the neck.

**Figure 5:**
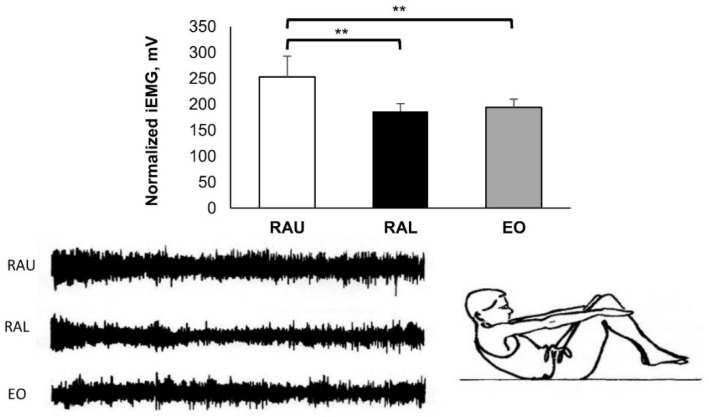
Hook lying with arms elevated forward and feet above the floor. Slow curl-up with rounded back to approximately 35–40°, with isometric contraction for 10 s. Group mean electric activity during the exercise. iEMG indicates surface electric activity. Raw EMG activity of the three muscles from one participant. RAU = rectus abdominis upper part; RAL = rectus abdominis lower part; EO = external oblique. Differences between muscles: ** *p* < 0.01.

Figure 5: Starting position: Hook lying with knees flexed (110°), feet did not touch the floor. Hands were elevated forward. Prior to the start of curl-up, the lumbar spine was pressed on the floor, and then a slow curl-up followed, with rounded back to approximately 35–40°. Head was flexed to chin-on-chest position with isometric contraction for 10 s.

**Figure 6:**
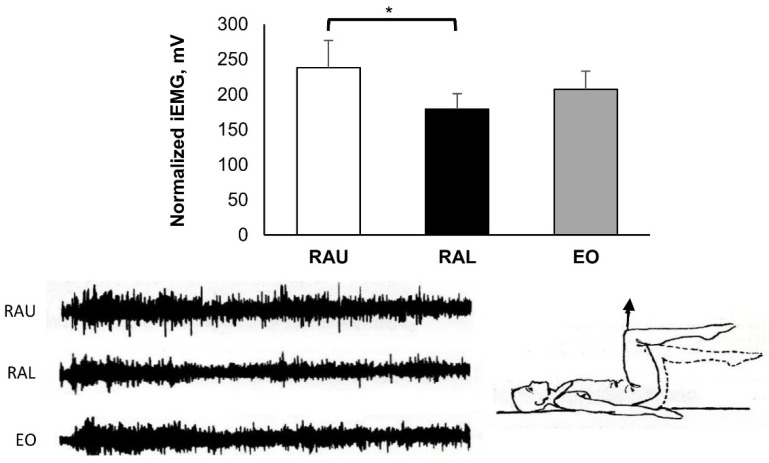
Hips and knees flexed 90°. Posterior pelvic tilt and lifting up the hips (see arrow) with isometric contraction for 10 s. Group mean electric activity during the exercise. iEMG indicates surface electric activity. Raw EMG activity of the three muscles from one participant. RAU = rectus abdominis upper part; RAL = rectus abdominis lower part; EO = external oblique. Differences between muscles: * *p* < 0.05.

Figure 6: Starting position: Lying position with hips and knees flexed 90°, above the floor. Posterior pelvic tilt and lifting the hips (gluteal region) from the floor through the direction of the knees. The arms were parallel with the body and pressed to the floor. The isometric contraction was approximately 10 s. This exercise had not been previously examined by means of EMG activity.

**Figure 7:**
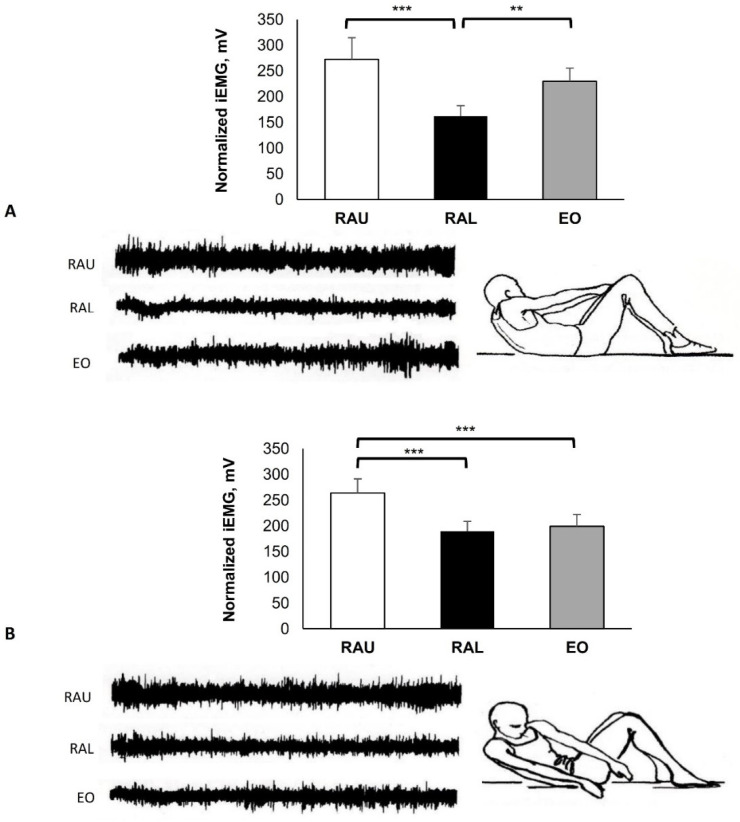
Supine position with bent knees and no stabilization of the lower limbs. Rotation of the trunk to (**A**) the left and (**B**) the right side of the body with isometric contraction for 10 s. The surface electrodes were placed on the right side of the external oblique muscle. Group mean electric activity during the exercise. iEMG indicates surface electric activity. Raw EMG activity of the three muscles from one participant. RAU = rectus abdominis upper part; RAL = rectus abdominis lower part; EO = external oblique. Differences between muscles: ** *p* < 0.01; *** *p* < 0.001.

Figure 7: Starting position: Hook lying with knees flexed (110°). The unsupported feet were flat on the floor. Curl-up with lateral rotation to the left side and with the right hand touching the lateral side of the left knee (A), and a lateral rotation to the right side with the left hand touching the lateral side of the right knee (B). The lumbar spine was flat on the floor.

**Figure 8:**
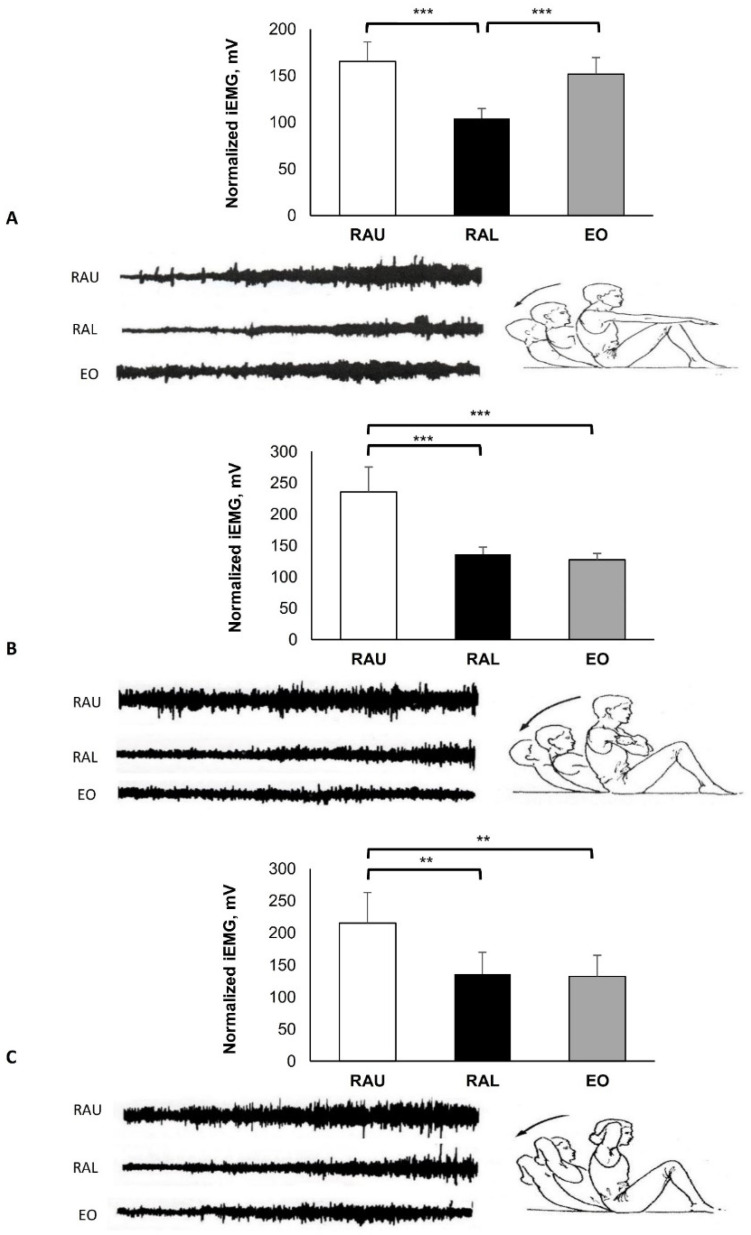
Eccentric exercises. From full trunk flexion with unsupported knees, and hands directed (**A**) forward, (**Β**) on the chest, and (**C**) behind the neck. Slow trunk extension with rounded back on the floor. Group mean electric activity during the exercise. iEMG indicates surface electric activity. Raw EMG activity of the three muscles from one participant. RAU = rectus abdominis upper part; RAL = rectus abdominis lower part; EO = external oblique. Difference between muscles: ** *p* < 0.01; *** *p* < 0.001.

Figure 8: Eccentric exercises. Starting position: Sitting (full trunk flexion) with unsupported bent knees and arms extended, attempting to touch the knees (A). This was performed with hands on the chest (B) and with the hands behind the neck (C). Slow, eccentric movement to the floor. This eccentric exercise started with a flexed head, chin on chest, and rounded back. The deceleration of the movement was carried out vertebrae by vertebrae (fall of the trunk); it started from the pressing of the lumbar spine, continued with the thoracic spine, and finally the cervical spine, which came into contact with the floor. In this way, the participants felt the contraction of the abdominal muscles.

### 2.5. EMG Recording and Data Analysis

Raw EMG signals were assessed with two miniature silver–silver chloride surface electrodes (Beckman Instruments, Inc., Fullerton, CA, USA) and were recorded on a calibrated Medelec MS 6 model (sweep velocity 10:1 Ms/dv), EMG signals at 2 kHz. Alcohol wipes were used for cleaning the surface of the skin before electrode placement. Bipolar electrodes were applied 6 cm below the xiphoid process for RAU, 6 cm distal of the umbilicus for RAL, and on each side of the middle line along the muscle direction. On the EO muscle, the electrode was placed on the right side from the center of the muscle, 3 to 4 cm besides the umbilicus, in a diagonal direction, coinciding with the muscle fibers. The EMG recordings were amplified by a factor of 1000, rectified, and integrated by calculating the area under the rectified curve, providing an appreciation of the total amount of surface electric activity (iEMG) during the exercise. The mean iEMG was calculated by dividing by the elapsed time for the five repetitions; the mean iEMG (MiEMG) for each exercise was used as a criterion for statistical analysis [23].

### 2.6. Statistical Analysis

All data were expressed as mean ± standard deviation (SD). The statistical analysis was undertaken using SPSS V.26.0 (SPSS Inc., Chicago, IL, USA). Data normality was verified with the Kolmogorov–Smirnoff test. One-way analysis of variance (ANOVA) with Bonferroni post-hoc analysis was applied to determine the differences between the muscle groups (RAU, RAL, and EO) for all exercises. Partial eta squared (η_p_^2^) effect sizes were calculated for the exercise type × muscle group interaction effects. An effect of η_p_^2^ = 0.2 indicated a small effect, =0.5 medium, and =0.8 large. The level of significance was set at *p* < 0.05.

## 3. Results

All the EMG activities were performed during various types of curl-up and sit-up exercises. The MiEMG and raw EMG data (from a selected subject) across all exercises for the three muscle groups are shown in Figure 3, Figure 4, Figure 5, Figure 6, Figure 7 and Figure 8. Figure 3 presents typical activity of the abdominal muscles with various leg movements. During the alternate up and down leg movement (scissors) (Figure 3A), there was a strong activation of the RAL (445.83 ± 64.83 mV), which showed higher activity compared with the RAU (285.70 ± 40.75 mV) and the EO (231.17 ± 37.94 mV) (*p* < 0.001), respectively. As seen in Figure 3B, the EO (163.90 ± 31.67 mV) was less activated in comparison with the RAU (250.67 ± 32.78 mV) and RAL (279.00 ± 32.59 mV) (*p* < 0.001), respectively. During bilateral straight leg raising and isometric contraction (Figure 3C), the RAL (271.17 ± 23.77 mV) showed higher EMG activity compared with the RAU (197.00 ± 31.84 mV) and the EO (148.93 ± 13.81 mV) (*p* < 0.001); and the RAU activity was higher in comparison with the EO (*p* = 0.011).

The dynamic sit-up movements (concentric and eccentric muscle contraction) (Figure 4) showed obvious dominant activity of the EO (221.60 ± 10.56 mV), which was higher compared with RAU (151.42 ± 10.59 mV) and RAL (144.57 ± 11.72 mV) (*p* < 0.001).

In the exercise shown in Figure 5, there was higher activity in the RAU (252.83 ± 39.93 mV), in comparison with the RAL (186.63 ± 14.66 mV; *p* = 0.002) and the EO (194.13 ± 15.97 mV; *p* = 0.004).

In the exercise shown in Figure 6, the behavior of EMG activity was completely different among subjects and the RAU (238.17 ± 38.76 mV) was higher compared with the RAL (179.10 ± 21.87 mV; *p* = 0.011).

During the left lateral rotation (Figure 7A) the activity of the RAU (272.67 ± 41.88 mV) was similar to the EO (229.90 ± 25.56 mV); while during the lateral rotation to the right (Figure 7B), the RAU (263.67 ± 27.63 mV) had higher activity in comparison with the EO (199.00 ± 22.98 mV; *p* < 0.001). It should be emphasized that the RAU and the RAL had similar activity, irrespective of whether the lateral rotation was performed to the right or the left.

During the eccentric exercises (Figure 8A), the EMG activity increased more at the end of the movement, when the subject approached the mat. Higher EMG activity was found in the RAU (165.43 ± 20.92 mV) compared with the RAL (104.00 ± 10.86 mV; *p* < 0.001). The EO (151.88 ± 17.78 mV) was more regular throughout the range of the movement and showed higher activity in comparison with the RAL (*p* < 0.001). Similar patterns were found in Figure 8B,C, where the RAU (235.67 ± 39.74 mV and 215.10 ± 47.79 mV, respectively) was higher, compared with the RAL (136.50 ± 10.97 mV, *p* < 0.001 and 135.83 ± 33.83 mV, *p* < 0.009, respectively) and the EO (127.17 ± 10.57 mV, *p* < 0.001 and 132.08 ± 32.82 mV, *p* < 0.006, respectively).

## 4. Discussion

The movements shown in Figure 3A–C are performed by the hip flexors, particularly by the iliopsoas, rectus femoris, and sartorius [24]. The contractions of these muscles increases lordosis in the lumbar spine, while the abdominal muscles have static action, stabilising the pelvis and preventing it from lateral or anterior rotation.

The bilateral lift of the legs 30 cm above the floor (Figure 3C) is an exercise involving two movements; first, a contraction of the hip flexors to lift the legs, and second, maintaining the lift by the isometric contraction of the abdominal muscles. In many exercises, the abdominal muscles have a stabilizing function, which facilitates the movement’s correct performance. To achieve this, the abdominal muscles need sufficient strength to be able to stabilize the pelvis (fixation of the pelvis). The weight of the lower limbs usually corresponds to about 40% of total body weight. The lower limbs are held above the floor by the contraction of the hip flexors, while the neutral position of the pelvis is achieved by the isometric stabilizing force of the abdominals, especially of the RAL. In the above exercises (Figure 3A–C), the high activity of the abdominal muscles is achieved at the expense of uneven load in the intervertebral discs, and this method is therefore not recommended for abdominal strengthening [25].

Dynamic sit-ups with a quick start (flexion concentric and extension eccentric) were performed through the full range of trunk flexion (Figure 4). Motor unit activity of the abdominals increased but to a comparatively low degree. This may be due to the quick start from the floor, where there was not enough time for the abdominal muscles to contract. Furthermore, it has been reported that support on the feet activates the hip flexors and reduces the activity of the abdominal muscles [10]. Nachemson [26] reported increased pressure on the intervertebral disc at the level of L3 during the execution of full sit-ups. This must be taken into consideration when training continues over many years. Rectus abdominis muscle activity was greatest in the early stages of trunk flexion and decreased as the range of motion became greater, more than 35–40°. The highest EMG activity was shown during the trunk flexion (concentric) and at a lower position during the extension of the trunk (eccentric). Andersson et al. [27] reported that during concentric muscle contraction, the activity of the abdominal muscles is 50% higher than during eccentric muscle contraction.

In the curl-up exercise shown in Figure 5, the abdominal muscles were activated more when the legs were above the floor. The upper and the lower body were lifted by the same muscle, and this could be the reason for the high activation of the abdominals. It is also possible that during this exercise the lower extremities and the hip flexor muscles, which participated during the sit-up, may be inactive due to the shortened position, so the abdominal muscles could be more activated. Similar results have been reported in previous investigations [28,29,30,31,32,33].

The exercise illustrated in Figure 6 showed differences in EMG activity between the subjects. This, combined with the increased EMG activity overall, may be due to participants’ fatigue. It was difficult for many subjects to stabilize the trunk and thereby activate their motor units for the whole duration of the exercise. The participants led their knees to the chest, instead of lifting them vertically upwards. The reasons might have been that they could not easily recruit their motor units, while reduced spinal flexibility and possibly tight low back muscles were more likely to block the pelvis tilt and lift the knees up. Therefore, prior to a test or training of the abdominal muscles, it is important to perform pre-exercises that include mind–muscle connection to ensure the correct execution of the movements.

In Figure 7, it can be seen that the RAU and RAL had similar activity during curl-ups with lateral rotation to the left and right, regardless of which direction the lateral rotation followed. This was expected, as the RAL remained still on the floor. The difference observed between the RAU and the EO only during the right lateral rotation may be due to variance of the participants’ trunk mobility. Our results are in agreement with previous studies with similar methodological approaches [34,35].

Eccentric exercise can be an effective way to improve reduced neuromuscular function (recruitment and synchronization of motor units) [36]. The eccentric exercise shown in Figure 8 has the advantage that participants are “forced” to feel the muscular contraction. Therefore, it can be used by untrained and overweigt individuals, as well as by those with poor muscular sensitivity. This technique is also considered to be a good proprioceptive exercise for strengthening the abdominal muscles.

### 4.1. Limitations

This study had several limitations. The sample size was small and there was a lack of electromyographic data for other muscles that are also responsible for the stability of the trunk (e.g., the internal oblique and the transversus abdominis). Also, the exercises were performed by each participant in the same order, which can result in potential order effect. Furthermore, only healthy male university students with a subspecialty in soccer were tested in the present study. This reduced possible bias but limited the generalizability of the findings.

### 4.2. Practical Applications

Our study was performed on young adults, but this does not exclude the fact that adherence to proper form for the exercises as described in this study must be applied in clinical and sport rehabilitation settings. Correct application of the exercises mentioned in this study can improve the performance of the trunk and back muscles, effective in the prevention and treatment of lower back pain. The findings of the present study will be a useful tool for training purposes, and will provide important information for more effective and preventive training programs.

## 5. Conclusions

The RAU and the RAL showed different EMG activation in some exercises, despite their being parts of the same muscle. However, sit-up exercises towards the knee activated almost equally the RAU and the RAL. Dynamic or isometric curl-ups performed through a partial range may be an effective method for strength training of the abdominal muscles, because they exert comparatively less strain on the lumbar spine. The methodological approach of this study and its findings suggest that future research should focus on the fact that stretching and pre-exercises are required before the implementation of any test or training for the abdominal muscles, and that spinal flexibility and the muscles surrounding the pelvis should be assessed. Proper abdominal muscle training is useful when seeking to activate the muscles within the physiological curvature of the spine.

## Data Availability

The data presented in this study are available on request from the corresponding author. The data are not publicly available due to privacy restrictions.
