# Expression of Concern: Mandroukas et al. Surface Electromyographic Activity of the Rectus Abdominis and External Oblique during Isometric and Dynamic Exercises. *J. Funct. Morphol. Kinesiol.* 2022, *7*, 67

**DOI:** 10.3390/jfmk11010052

**Published:** 2026-01-26

**Authors:** Journal of Functional Morphology and Kinesiology Editorial Office

**Affiliations:** MDPI, Grosspeteranlage 5, 4052 Basel, Switzerland; jfmk@mdpi.com

With this notice, the Journal of Functional Morphology and Kinesiology Editorial Office alerts the readers to concerns related to this article [1]. Following publication, concerns were raised with the Editorial Office regarding the ethics approval information presented in this publication, and the extent to which this information adheres to national legislation. The Editorial Office, with the involvement of the Editor-in-Chief, has decided to issue this Expression of Concern while an investigation is being conducted. The authors and their affiliated institution have been contacted to assist with the investigation.

Once the investigation is complete, the Editorial Office will provide an update on the situation and announce any post-publication modifications deemed necessary, as per MDPI′s Update to Published Papers policy (https://www.mdpi.com/ethics#_bookmark30, accessed on 12 December 2025).
